# Adverse health effects of low levels of perceived control in Swedish and Russian community samples

**DOI:** 10.1186/1471-2458-7-314

**Published:** 2007-11-02

**Authors:** Johanna Lundberg, Martin Bobak, Sofia Malyutina, Margareta Kristenson, Hynek Pikhart

**Affiliations:** 1 Department of Medical and Health Sciences, Linköping University, Sweden; 2 International Institute for Society and Health, Department of Epidemiology and Public Health, UCL, London, UK; 3 Institute of Internal Medicine, Russian Academy of Medical Sciences, Novosibirsk, Russia

## Abstract

**Background:**

This cross-sectional study of two middle-aged community samples from Sweden and Russia examined the distribution of perceived control scores in the two populations, investigated differences in individual control items between the populations, and assessed the association between perceived control and self-rated health.

**Methods:**

The samples consisted of men and women aged 45–69 years, randomly selected from national and local population registers in southeast Sweden (n = 1007) and in Novosibirsk, Russia (n = 9231). Data were collected by structured questionnaires and clinical measures at a visit to a clinic. The questionnaire covered socioeconomic and lifestyle factors, societal circumstances, and psychosocial measures. Self-rated health was assessed by standard single question with five possible answers, with a cut-off point at the top two alternatives.

**Results:**

32.2 % of Swedish men and women reported good health, compared to 10.3 % of Russian men and women. Levels of perceived control were also significantly lower in Russia than in Sweden and varied by socio-demographic parameters in both populations. Sub-item analysis of the control questionnaire revealed substantial differences between the populations both in the perception of control over life and over health. Logistic regression analysis revealed that the odds ratios (OR) of poor self-rated health were significantly increased in men and women with low perceived control in both countries (OR between 2.61 and 4.26).

**Conclusion:**

Although the cross-sectional design does not allow causal inference, these results support the view that perceived control influences health, and that it may mediate the link between socioeconomic hardship and health.

## Background

In the second half of the 20^th ^century, health status has declined in Central and Eastern Europe, resulting in decreasing life expectancies, while health status in Western Europe has improved [1-3]. Among the former communist countries, Russia experienced the most dramatic decline in life expectancy, particularly during the 1990s, resulting in a near 20-year difference to Swedish men [4]. As an illustration, life expectancy in Russia was 58.9 years for men and 71.8 years among women in 2001, comprising 3 million more middle-age deaths than if rates would be based on 1991 mortality rates [4]. Also, measures of self-rated health and physical functioning are significantly lower compared to Swedish rates, for both men and women [5,6]. These differences have not been explained by environment or lifestyle factors, and have generated an interest in other risk factors, such as psychosocial factors [1]. The general impact of psychosocial factors on health has been illustrated by Kristenson et al [7] who have shown, on the example of the high CHD mortality rates in Lithuania, how levels of vital exhaustion (a measure of extended physical and mental fatigue) and depression were substantially higher in Lithuania than among Swedish middle-aged men.

An important psychosocial factor, though not well-explored in terms of East-West comparisons, is perceived control, which represents individuals' perceptions of the magnitude of power over their own lives [8-13]. This concept is closely related to other concepts of individual psychological characteristics, such as coping ability, mastery, self-esteem and internal locus of control, which all relate to peoples learned outcome expectancies [14]. Measures of perceived control have proved to be a robust predictor of mental and physical well-being and of all-cause mortality [15], and perceived control has been shown to explain a substantial part of SES differences in mortality in cohort studies [16,17]. Low control over life was further found to account for the negative impact of low social position on health, while good family relations protected against poor health [16]. Carlson [6] points to the fact that a sense of life control is important for people's self-perceived health in almost every European country, but that in the former communist countries, control levels are generally lower than in the west. In cross-sectional studies, low perceived control has been shown to be related to low self-rated health as an independent factor in Eastern Europe [12,13]. Deprivation and low perceived control were here suggested as important mediators in the relation between social circumstances and health.

Furthermore, the East-West health gap seems to follow the same gradient that has repeatedly been found across the social spectrum in almost every modern society, stating that the lower the socioeconomic status (SES), the worse the health status [1,18,19]. Not only do relative differences *between *countries seem to matter, but also relative differences *within *each population are important. Just as the East – West differences, these variations in life expectancy and health can only partly be explained by lifestyle factors, such as smoking, alcohol intake, poor diet and poor exercise habits [1,12,18,20]. Psychosocial factors have been proposed to explain at least part of social gradients in health in western Europe and United States [11,21,22]. Gilmore, McKee & Rose [23] demonstrated that SES including a poor material situation as well as psychosocial factors, among them low control over life, were independent determinants of self-rated health in Ukraine.

The vast societal changes in post-communist countries since the beginning of the 1990's resulted in growing inequalities in income distribution along with material deprivation, and in the collapse of social institutions [6,12,24-26]. The health impact of these changes, emanating from the relationships between individual needs, the social structure and the psychosocial environment, should be of importance for further studies. Earlier findings imply that low levels of control are likely to contribute to the poor health situation following transition in Russia [12]. We therefore see perceived control as an important focus for East-West comparative studies. Comparing populations in different political and cultural settings, such as Sweden and Russia, could provide valuable knowledge on the intrusiveness of psychosocial factors on health, as well as to shed light on the importance of relative position and social status within populations.

Our aim is to investigate levels in the perception of life control among men and women in community samples of Swedish and a Russian population, and to assess whether these measures are associated with self-rated health in each of the studied communities. We also aim at identifying socio-demographic differences related to perceived control, within and between the populations, and to explore differences in answer patterns to the perceived control questionnaire.

## Methods

### Populations and subjects

This paper uses data from two related studies, both originally designed to enable comparative analyses by a common sampling strategy and a common study protocol for a core battery of parameters: The Swedish LSH study (Life conditions, Stress, and Health) and the HAPIEE study (Health, Alcohol and Psychosocial Factors in Eastern Europe) [27]. Both studies are still ongoing, and follow-up data collections are currently in process. The current analysis is based on the baseline data collection.

For the Swedish LSH Study, the baseline data were collected during 2003–2004. Participants were 1007 men and women aged 45–69 years in 2003, stratified by 5-year age groups, and belonging to any of the catchment areas of 10 primary health care centres in southeast Sweden (response rate 62%). Participants fulfilling these requirements were randomly selected via the National population register. Data collection at baseline included self-reported data via postal questionnaires, and measures of blood pressure, anthropometrics and blood sampling during a visit to a local clinic. Exclusion criteria were serious disease and difficulties in understanding the language. The study population is nationally representative in terms of age, civil status and educational level.

The Russian data come from the baseline phase of the Russian part of the HAPIEE study in 2002–2005. A sample of men and women 45–69 years old, stratified by gender and 5-year age groups, was randomly chosen from local population register of Novosibirsk town, and selected individuals were invited to participate in the study. The data analysed in this report were collected by a structured questionnaire and by the examination at local clinics; 9231 men and women aged 45–69 years participated in the baseline examination (response rate 61%). The study population is representative for Russian urban population in terms of age, sex and educational level.

### Measurements

The structured questionnaires in both countries contained a common set of identical core parameters that cover a broad amount of topics, such as socioeconomic status, a section on psychosocial measures, health behaviours, self-rated health and diagnosed illnesses. All questionnaires were administered by mail in Sweden, while in Russia participants needed to visit the clinic in order to fill in the questionnaires. Correct wording was checked by translating both, Swedish and Russian, questionnaires back into English.

A score of *perceived control *was based on agreement or disagreement with eleven statements adapted from the Whitehall II Study [9], MacArthur Foundation programme on Midlife development [10] and the New Barometer studies [12,13]. They are similar to the questions on perceived constraints used and extensively validated by Lachman and Weaver in the US [11]. Lachman [28] reviewed use of sense of control measures in midlife studies. Use of this instrument in Russia and six other post-communist countries was validated by Bobak et al [13]. Exact wording of used questions is presented in one of the tables. Items 2, 3 and 4 are generally seen as representing "control over health" while the other items represent "control over life". The subjects were asked to what extent they agree or disagree with the statements. The answers were recorded at a six-point scale (coded as 0–5). Scores were calculated if a minimum of 9 out of 11 questions contained valid answers. If less than 11 valid answers, the score was divided by the number of valid answers and then multiplied by 11. The final score ranged between 0 (no control) and 55 (maximum control). Internal consistency as assessed by Cronbach's alpha was 0.66 and 0.71 for Swedish men and women, and 0.64 and 0.63 for Russian men and women. We have also constructed two subscales, control over health and control over life, and assessed their internal consistency. Internal consistency of subscales was not better than of the whole scale: Cronbach's alpha for health control ranged between 0.60 and 0.66 and for life control between 0.60 and 0.69 when separately calculated for Swedish and Russian men and women.

*Self-rated health *[29] was assessed by a standard single question with answers on a 5-point scale. For the analysis, the dichotomized outcome was defined as the top two categories representing good health, while poor health was defined as the bottom three categories.

*The characteristics *used in different stages of analysis included: civil status, education, body mass index (BMI), total cholesterol, blood pressure measurement and smoking. Civil status was classified into 4 categories: married/cohabiting, single, divorced and widowed. Education was classified into 3 categories: primary or less, secondary/vocational and completed university. BMI was calculated from measures obtained at the clinical investigation. Serum concentrations of total cholesterol were obtained from blood collected at the clinical examination. Measurements of blood pressure were collected also at the clinical examination. Smoking was assessed through four options: never smoked, have quit smoking, smoke less than 1 cigarette/day, smoke regularly at least 1 cigarette/day, where the latter two were categorized into "regular smoker".

### Statistical analysis

Distributions of background factors and self-rated health were calculated for both populations, and were also stratified by sex within the two countries. For comparative purposes, means and distributions of perceived control were calculated for each country separately. Logistic regression was used to analyse the associations between psychosocial factors and self-rated health, firstly controlling for age only, and secondly controlling for age, education, civil status, obesity (in terms of BMI), blood pressure, cholesterol levels, and smoking. All analyses were conducted separately for men and women for each of the study populations. A p-value of < 0.05 was regarded as significant.

## Results

### Demographics

Descriptive characteristics of the two samples are presented in Table 1. The two samples are similar in terms of age groups and with regards to the proportion of women and men. Three times as many Russian women (14.5%) were divorced, compared to Russian men (5.5%). Among the Swedish women and men, rates were lower and more equal (8.8% vs. 6.3%). While 21.3 % of Russian women were widowed, this was the case for just 4.0 % of Russian men. For Swedish men and women, rates were 0.8 % and 5.9 %. Russians reported to a higher extent a university degree than did Swedes (28.9% vs. 21.2%), while also presenting lower rates of primary education only (10.3% vs. 35.6%). These differences remain within both sexes when comparing the populations. 49.8 % of Russian men reported smoking regularly, compared to just 10.5 % of Russian women. Among the Swedes, about a fifth of the population reported regular smoking (same share for both sexes). Mean BMI (not presented in table) values ranged from 26.5 to 30 with Russian women counting the highest mean level.

**Table 1:** Descriptive characteristics of LSH and HAPIEE participants

	**Sweden**			**Russia**		
	Total (n = 1007)	Men (n = 502)	Women (n = 505)	Total (n = 9231)	Men (n = 4201)	Women (n = 5030)

**Age group**	%	%	%	%	%	%
45–49	19.7	19.7	19.6	17.0	15.8	18.0
50–54	20.2	20.1	20.2	19.6	19.9	19.3
55–59	20.4	20.7	20.0	21.7	21.8	21.6
60–64	19.8	19.7	19.8	19.2	19.5	19.0
65–69	20.1	19.7	20.4	22.5	22.9	22.1
						
**Civil status**						
Single	6.6	6.5	6.7	3.8	2.7	4.7
Married/cohabiting	82.5	86.3	78.6	72.3	87.8	59.4
Divorced	7.5	6.3	8.8	10.5	5.5	14.5
Widowed	3.4	0.8	5.9	13.4	4.0	21.3
						
**Education**						
Primary	35.6	36.4	34.8	10.3	11.2	9.5
Secondary	43.2	43.1	43.4	60.8	56.8	64.1
University	21.2	20.5	21.7	28.9	32.0	26.4
						
**Smoking**						
Non-smoker	42.4	38.1	46.7	58.1	25.7	85.2
Ex-smoker	38.6	42.7	34.4	13.6	24.5	4.3
Regular smoker	19.0	19.2	18.9	28.3	49.8	10.5
						
**Self-rated health**						
1 (very good)	8.2	8.7	7.6	0.2	0.2	0.2
2	24.0	27.4	20.6	10.1	15.5	5.6
3	41.0	41.6	40.3	67.4	67.5	67.2
4	22.5	19.5	25.5	20.8	15.8	24.9
5 (very poor)	4.4	2.8	6.0	1.6	1.0	2.1

### Self-rated health

Self-rated health was assessed by a standard single question with answers on a 5-point scale, with the top two categories set as representing "good health". Rates for good health in the Swedish population were clearly higher than in the Russian population (32.2% vs. 10.3%). Almost none of the Russians reported the top alternative, "very good health" (0.2 %), while 8.2% of Swedes did this. Swedish women report good health to a lesser extent than do Swedish men (28.2% vs. 36.1%), and Russian women score not only lower than Russian men (5.8% vs. 15.7%), but also present the lowest scores of all four groups. In general, women in both populations show a tendency to report scores at the lower end of the scale more than men do.

### Perceived control

**Figure 1:**
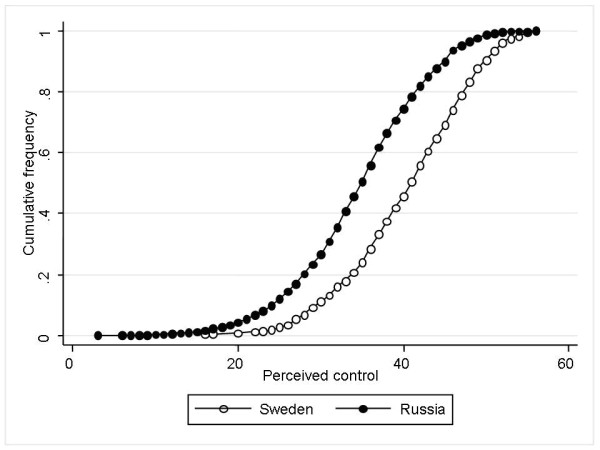
Cumulative frequency of perceived control scores in Sweden and Russia.

**Figure 2:**
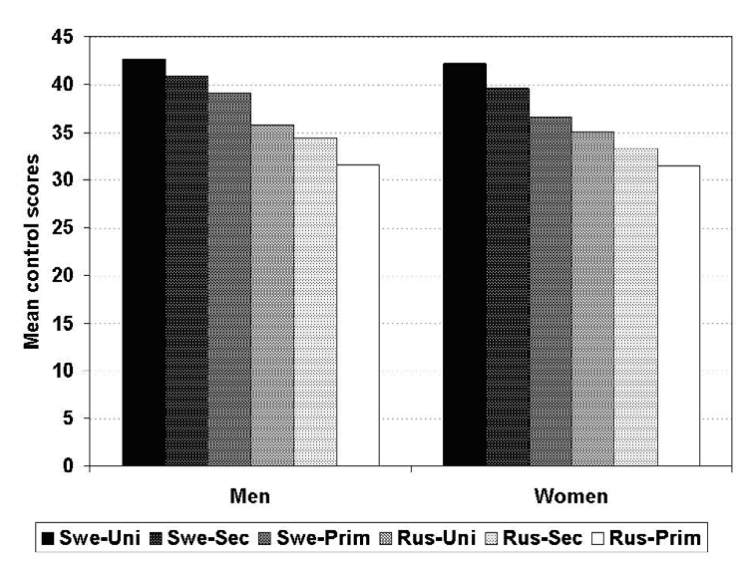
Mean control score per level of education for men and women in Sweden and Russia.

The mean control scores were higher in the Swedish population. For Swedish men, the mean control score was 40.6, while for Russian men it was 34.5 (Table 2). For Swedish women, the mean score was 39.0, compared to 33.6 for Russian women (for both sexes p < 0.001 for differences in control scores between 2 countries). Figure 1 shows cumulative frequency of perceived control score. Lower control scores among Russians can be seen across whole sample distribution. Control levels showed a linear relation to educational status within both populations, but the scores also formed a social gradient across the countries, as the highest scores were seen with highly educated Swedes while the lowest scores were found among Russians with low education (Figure 2). Widowed women had the lowest control score among the total Swedish population, but their score was still higher than the total mean score of Russian women. Widowed men had the lowest control score in the Russian sample. Control levels were also, in general, decreasing with higher age, except for Swedish men who instead seem to increase their control levels up to 55–59 years of age before declining.

**Table 2:** Age, sex, education and civil status specific scores of perceived control

	LSH – Sweden				HAPIEE – Russia			
	Men		Women		Men		Women	

**Control**		Alpha		Alpha		Alpha		Alpha

Sum score -mean	40.56	0.66	39.00	0.71	34.51	0.64	33.59	0.63
-SD	7.20		8.06		8.00		7.94	
Age								
45–49	40.32	0.71	40.24	0.70	36.74	0.60	35.45	0.63
50–54	40.77	0.67	41.13	0.73	35.45	0.59	34.88	0.63
55–59	42.67	0.58	39.49	0.71	34.79	0.64	33.60	0.65
60–64	40.67	0.69	37.61	0.69	33.98	0.65	32.76	0.58
65–69	38.17	0.66	36.53	0.67	32.34	0.64	31.64	0.61
p*	*0.001*		*< 0.001*		*< 0.001*		*< 0.001*	
Education								
Primary or less	39.10	0.61	36.61	0.66	31.57	0.74	31.53	0.69
Secondary/Vocational	40.89	0.65	39.53	0.70	34.38	0.60	33.28	0.63
University	42.60	0.73	42.23	0.73	35.79	0.62	35.06	0.61
p*	*< 0.001*		*< 0.001*		*< 0.001*		*< 0.001*	
Civil status								
Married/cohabiting	40.91	0.67	39.65	0.72	34.81	0.63	34.26	0.62
Single	37.19	0.78	39.46	0.63	33.18	0.72	32.65	0.63
Divorced	40.22	0.53	37.19	0.72	33.17	0.63	33.09	0.65
Widowed	39.48**	0.61	34.12	0.71	30.77	0.68	32.26	0.64
p*	*0.07*		*0.003*		*< 0.001*		*< 0.001*	

Alpha = Cronbach alpha

* p-value for one-way ANOVA

** only 4 individuals

### Analysis of individual control items

Country and sex specific comparisons of each of the control items are presented in Table 3. There were substantial differences (diff > 0.50) in both health-related control items and life control items, with nearly all items showing higher control in Sweden than in Russia. The largest difference between the populations altogether is found among men and women on item 4 – "There are certain things I can do for myself to reduce the risk of getting cancer" – where the Russians are highly negative. They are also more negative when it comes to their chances of reducing the risk of a heart attack (item 3). However, the Russians (especially the women) are more positive than the Swedes on item 2 – "Keeping healthy depends on things that I can do". Further, Russian men and women believe to a substantially lower extent than do Swedes that they will have more positive than negative experiences in the future. The Russians also report lower scores in relation to often being treated unfairly, and that the past ten years have been full of changes without their knowing of what will happen next. However, Russians report that they feel in control at home almost just as much as Swedes, and they seem to find more meaning in their daily life than do Swedes. The lowest mean rates in both populations and for both sexes on any item were found for control over life events (item 5), followed by control over the future during the past 10 years (item 8).

**Table 3:** Country and sex specific comparison of control items

	Men				Women			
Item	Swe	Rus	Diff (95% CI)	p-value for difference	Swe	Rus	Diff (95% CI)	p-value for difference
					
	mean	mean			mean	mean		

Item 1 At home, I feel I have control over what happens in most situations	4.38	4.23	0.15 (0.04,0.26)	0.007	4.32	4.25	0.07 (-0.04,0.18)	0.21
Item 2 Keeping healthy depends on things that I can do	*4.03*	*4.09*	-0.06** (-0.18,0.06)	*0.32*	*3.81*	*4.13*	-0.32** (-0.43,-0.20)	*< 0.001*
Item 3 There are certain things I can do for myself to reduce the risk of a heart attack	4.37	3.38	0.99 (0.84,1.14)	< 0.001'	4.37	3.55	0.82 (0.68,0.96)	< 0.001
Item 4 There are certain things I can do for myself to reduce the risk of getting cancer	3.62	2.13	1.49 (1.32,1.67)	< 0.001	3.50	2.28	1.22 (1.05,1.39)	< 0.001
Item 5* I feel that what happens in my life is often determined by factors beyond my control	2.21	1.81	0.40 (0.25,0.54)	< 0.001	1.94	1.60	0.34 (0.20,0.47)	< 0.001
Item 6 Over the next 5–10 years I expect to have many more positive than negative experiences	3.91	3.02	0.89 (0.73,1.05)	< 0.001	3.96	3.11	0.85 (0.69,1.01)	< 0.001
Item 7* I often have the feeling that I am being treated unfairly	3.95	2.88	1.07 (0.92,1.22)	< 0.001	3.76	2.66	1.09 (0.93,1.25)	< 0.001
Item 8* In the past ten years my life has been full of changes without my knowing what will happen next	2.68	1.90	0.78 (0.61,0.95)	< 0.001	2.19	1.69	0.50 (0.34,0.67)	< 0.001
Item 9* I very often have the feeling that there's little meaning in the things I do in my daily life	*3.33*	*3.78*	-0.46** (-0.61,-0.31)	*< 0.001*	*3.25*	*3.48*	-0.23** (-0.39,-0.07)	*0.005*
Item 10* I sometimes feel as if I've done all there is to do in life	3.86	3.59	0.28 (0.12,0.43)	< 0.001	3.87	3.43	0.44 (0.28,0.60)	< 0.001
Item 11* I gave up trying to make big improvements or changes in my life a long time ago	4.18	3.68	0.50 (0.35,0.65)	< 0.001	4.02	3.41	0.61 (0.44,0.77)	< 0.001

*item used in reverse order in the table to have 0 as low control and 5 as high control

** items in italics are those with higher control among Russian subjects

### Associations between perceived control and self-rated health

Table 4 shows results from logistic regression in two stages of adjustment. In the age-adjusted analyses, the OR of poor self-rated health was increased in groups with low perceived control in all four subgroups (p-value for test for trend in ORs < 0.001 in all 4 groups). Controlling for covariates reduced the odds ratios (3.45 and 4.26 for Swedish men and women, respectively, and 2.61 and 3.74 for Russian men and women, respectively) but the associations remained highly significant. Although the effect of perceived control slightly differs for Sweden and Russia, the statistical interaction between country and control score was not significant either for men or women.

**Table 4:** The association between self-rated health and perceived control in both samples (country-specific quartiles)

	Age-adjusted		Fully adjusted*	
	Sweden	Russia	Sweden	Russia

Men				
1Q (low control)	3.95 (2.21–7.07)	2.87 (2.19–3.75)	3.45 (1.77–6.70)	2.61 (1.98–3.45)
2Q	3.19 (1.81–5.61)	1.57 (1.24–1.97)	2.24 (1.20–4.20)	1.46 (1.16–1.85)
3Q	1.84 (1.09–3.10)	1.17 (0.95–1.45)	1.51 (0.85–2.70)	1.15 (0.93–1.43)
4Q (high control)	1	1	1	1
P for trend**	*< 0.001*	*< 0.001*	*< 0.001*	*< 0.001*
				
Women				
1Q (low control)	5.44 (2.83–10.47)	4.30 (2.87–6.44)	4.26 (2.07–8.78)	3.74 (2.48–5.64)
2Q	5.11 (2.78–9.38)	2.72 (1.98–3.76)	5.06 (2.59–9.90)	2.53 (1.83–3.51)
3Q	3.22 (1.82–5.68)	2.08 (1.51–2.87)	3.42 (1.83–6.41)	2.00 (1.45–2.78)
4Q (high control)	1	1	1	1
P for trend**	*< 0.001*	*< 0.001*	*< 0.001*	*< 0.001*

* adjusted for age, education, civil status, BMI, smoking, blood pressure and cholesterol

** test for linear trend in log-odds of poor self-rated health (following Mantel [48])

Q = quartile

## Discussion

This comparison of two community samples from Sweden and Russia represents two contrasting political and socio-cultural environments. We found that Russians reported lower perceived control on most items, and that they also reported poorer self-rated health than the Swedes. In both countries, perceived control was associated with poor self-rated health.

### Control and self-rated health

The findings of low perceived control and self-rated health in Russia, as compared to Sweden, is in line with an earlier study by Carlson [30]. However, to our knowledge, no other studies have examined perceived control from an East-West perspective. Furthermore, while the Carlson study used a one-item measure of "life control", graded from 1 to 10, the measure of perceived control used here is a more complex instrument counting 11 sub-items.

We noticed differences in control levels between socio-demographic groups within both populations, most visibly so in women who generally reported lower levels of control than men. Our findings of lower perceived control among the lower educated, the elderly and single people in both populations are in line with earlier reports on social status and psychosocial factor prevalence [7,12,13,31,32]. Furthermore, assumptions of a social gradient over the continent [1] was supported by our data, as perceived control was lowest among men and women with the lowest level of education in Russia, and highest among people with high education in Sweden (Figure 2). We used education as an indicator for social position although it is possible that other indicators, such as income, could be strongly related to control. However, unfortunately, data on income were not available in this analysis.

We also found significant associations between perceived control and self-rated health in both sexes in both populations. This is again in line with the studies by Carlson [30] and Bobak et al. [13], who found that life control had a similar effect on self-perceived health in different countries. Although Carlson concluded that control alone is unlikely to explain the East-West health divide, all these results suggest that that perceived control does have an evident impact on health, and that further studies on this aspect of individuals' lives are required.

### Differences in control by age and sex

Control decreased with age, but there were differences between the populations. There was a linear decrease for both men and women in Russia, but not in Sweden where the decrease for men starts after the age of 60, and in Swedish women after the age of 55. This suggests a faster decline in Russia, which is in line with earlier studies on self-rated health and physical functioning in Russia and Sweden [5]. Until the age of 55–59 years in men and 50–54 years in women, the prevalence of poor health were similar in the two populations, but after those ages, poor health increased rapidly in Russia. A similar pattern was found by Andreev et al [2] on healthy life expectancy in Russia and Western Europe.

Russian women stand out as suffering from worse health conditions compared to all other groups in our analysis. Their levels of poor self-rated health are significantly worse than those of any other group. Suggesting that health levels may depend on – or reflect – different reactions to adversities among the sexes in Russia, Andreev et al. [2] imply in their study on health expectancy in Russia, that although the premature male mortality in Russia is the most striking feature of the nation's health development, there also appears to be a substantial burden of ill-health among women – "men die while women suffer".

### Sub-item analysis of perceived control

Regarding the sub-item analysis of perceived control, according to traditional views on psychometrics, there may be reasons for keeping sub-items together as an instrument and not analyze these questions separately [33]. However, since earlier studies have settled for one-item control questions [6] or have used the perceived control instrument without any sub-item analysis [12,13], one of our aims was to shed more light on what differences may exist on sub-item level while having access to several control questions. This can be seen more as an explorative analysis rather than an actual confirmative study.

The whole concept of perceived control is closely related to the discussion of locus of control, and whether it is external or internal. Locus of control is usually described as the tendency in an individual to perceive a causal relationship between his/her own behaviour and what happens to him. Internal locus of control places the agent in an active role – the individual can take action over their own lives – while an external locus of control means that power over life lies outside of one's authority [34,35]. The perceived control instrument is designed to capture aspects of internal locus of control rather than that of an external locus [10]. A study by Leinsalu [34] used measures of locus of control to study differences in self-rated health by various dimensions of the social structure in Estonia. Leinsalu found that locus of control was strongly related to poor self-rated health, and that external locus of control was more prevalent among women but had a stronger association with poor self-rated health among men. However, to our knowledge, there are no studies that have investigated internal locus of control in an East-West perspective.

There were no substantial differences in the reporting of feeling in control at home between the populations. It is possible that this reflects the fact that the home environment is not the major source of psychological distress. As for the health-related questions (items 2–4), it is somewhat contradictory that the Russians scored higher than the Swedes in believing that "keeping healthy" depends on things they can do, while on items 3 and 4 scoring immensely low on their options of preventing cancer or heart attack. Perhaps "keeping healthy" is associated with an unspecific "health" outcome, possibly including conditions such as obesity or injury, whereas cancer and heart attack may be seen as external threats, impossible to overcome. If so, this should be a logic reaction, considering the high death rates in Russia, especially from heart disease [1,4]. Russians also scored better than the Swedes on the item regarding meaning in daily life; one could speculate that this difference may reflect the influence of collectivism that has prevailed since socialist days whereas Sweden in comparison since long is a secularized and individualized society.

### Learned helplessness – another aspect of control measures

The fact that more Swedes than Russians in our populations think that the next 5–10 years will give them more positive than negative experiences, while the Russians to a higher extent than Swedes believe that they are often being treated unfairly, could probably be seen as reflecting differences in the respective political systems of the populations, with differences in actual options of taking action and proposing changes [24-26], but it could also be a sign of learned expectation levels [36]. Several studies have shown that people in lower socioeconomic positions may experience psychosocial strain to a higher extent than those better off [7,31,32,37]. Different patterns of socialisation, when people notice that there is no relationship between their responses and the outcome, could lead to fatalism and expectations of having low prospects for the future, as well as inherited or learned coping strategies [15,36,38,35,14,39]. It is plausible that these coping mechanisms could also apply in whole populations; this could partly explain why the Russians score so low on the control items mentioned above. This is also consistent with the fact that the lowest mean rates for both populations on any item were found for control over life events, followed by control over the future during the past 10 years, both again possibly reflecting the uncertainties of societal changes and individual attitudes towards these.

### Psychosocial resources and intervention strategies

Reduction of social polarisations and increasing capacity from social environment to enhance positive outcome expectancies is what is aimed at in international and national targets for health e.g. the basis for the Ottawa manifest with its focus on health oriented policies and arena perspectives [40]. Above reduction of material deprivation, interventions in terms of empowerment strategies that build on the ambition to enhance individual chances of developing positive expectancies of the future, hope, self esteem and trust are fundamental [41]. The European Network of Health Promoting Agencies noted positive side effects in a project aiming at behavioural change, where people involved in planning and implementing the project reported increased levels of social integration and assertiveness [42].

### Methodological issues and limitations of the study

There are several problems with a study of this type. First, temporality of the association between perceived control and self-rated health is hard to establish in cross-sectional data. Prospective studies are needed to further validate these associations. Although two prospective studies found association between perceived control and mortality [16,17], a reverse causation of the relation between control and self-rated health cannot be excluded. As both the LSH and the HAPIEE projects are originally designed as prospective studies, we will revisit this analysis when follow-up data are available and look at the temporal associations.

Second, there is the general problem with validity of self-reported measures. Kristenson [35] has pointed out that a correlation between self-reported psychological measures and self-reported health could merely reflect an overlap between these measures, with results instead depending on some other common factor. While there are some studies that have suggested that self-rated health may partly reflect a sense of control [12,43], most studies have continued to use them as non-interfering variables in order to be comparable with earlier research.

To account for possible overlap between control and self-rated health, we have repeated the analysis with a control scale excluding three health-related items (items 2, 3, 4; see Table 3 for the exact wording of the items). The results did not substantially change when we excluded these three items although we can say that the results of the effects of this new control scale on self-rated health are now very similar in all four gender-country specific groups. In the fully adjusted analyses, the OR of poor self-rated health was increased in groups with low perceived control in all four subsamples (p-value for test for trend in ORs < 0.001 in all 4 subsamples). Those in the lowest quartile of control were approximately three times more likely to report poor self-rated health than those in the highest quartile of control (3.37 and 3.51 for Swedish men and women, respectively, and 3.28 and 3.41 for Russian men and women, respectively).

Third, chronic disease is a possible confounder in the relationship between control and health because an existing disease can reduce the feeling of control and self-perceived health. To account for this possible effect, we have conducted additional analysis excluding people who reported myocardial infarction, angina and stroke in the past. Results from our original analysis and new results were virtually same. This suggests that chronic disease (although expressed only in terms of cardiovascular disease) was not major confounding factor in this analysis

Fourth, psychosocial determinants of self-rated health may differ by age group. We believe that we have included the relevant age groups for this kind of study. Siegrist & Marmot [44] refer to the fact that midlife is the period of life, after the first year of life, during which social inequalities in health manifests themselves most strongly. This is in line with our data selection, comprising data on men and women aged 45–69 in Sweden and Russia.

Fifth, the HAPIEE sample possibly suffers from a health selection due to the fact that the study took part in a clinic. This is reflected by a better than expected levels of physical functioning (unpublished data), compared to data collected previously [5]. However, this bias would lead to overestimation of both control and health, and result in underestimation of the differences between Sweden and Russia.

The mode of administration of the questionnaires differed between countries, with self-reports in Sweden and interviewer-administered in Russia. There is evidence that using an interviewer may lead to under-reporting depression [45] or other self reported health outcomes [46,47] but we found higher levels of poor self-reported health and lower scores of control in Russia.

Sixth, non-response bias should also be considered. In general, people who participate in health surveys are healthier than those who do not. Thus, the levels of poor self-rated health in our study are probably underestimated. However, assuming that the differences between respondents and non-respondents were similar in both countries, the comparisons between the populations are valid, even if the absolute prevalence rates were underestimated (difference in response rates in both samples is approximately 1%). The non-response bias should not affect the association between self-rated health and perceived control within the study sample.

## Conclusion

In conclusion, levels of both perceived control and self-rated health are lower in Russia than in Sweden, and this is likely to reflect the differences in health status and in the social and psychosocial environments between the two countries. Within both populations, perceived control was strongly associated with self-rated health. Although the cross-sectional design does not allow causal inference, these results support the view that perceived control influences health and that it may mediate the association between socioeconomic hardship and health.

## Competing interests

The author(s) declare that they have no competing interests.

## Authors' contributions

JL participated in the acquisition and analyses of data, and drafted the manuscript. HP and MB participated in the design and coordination of the HAPIEE study, conducted analyses and helped draft the manuscript. SM participated in the design and coordination of the HAPIEE study and helped draft the manuscript. MK participated in the design of the LSH study, acquisition and analyses of data, and helped draft the manuscript. All authors read and approved the final manuscript.

## Pre-publication history

The pre-publication history for this paper can be accessed here:


